# Daily consumption of ready-to-use peanut-based therapeutic food increased fat free mass, improved anemic status but has no impact on the zinc status of people living with HIV/AIDS: a randomized controlled trial

**DOI:** 10.1186/s12889-015-2639-8

**Published:** 2016-01-04

**Authors:** Adama Diouf, Abdou Badiane, Noël Magloire Manga, Nicole Idohou-Dossou, Papa Salif Sow, Salimata Wade

**Affiliations:** 1 Laboratoire de Nutrition, Département de Biologie Animale, Faculté des Sciences et Techniques, Université Cheikh Anta Diop de Dakar, Dakar, BP 5005 Dakar - Fann Sénégal; 2 Service des Maladies Infectieuses, Centre Hospitalier National Universitaire de Fann, Dakar, Sénégal

**Keywords:** HIV/AIDS, Therapeutic food, Fat free mass, Anemia, Zinc, Senegal

## Abstract

**Background:**

Food insecurity in sub-Saharan Africa and malnutrition constitute the main obstacles for successful treatment of people living with HIV/AIDS (PLWH). The aim of this study was to assess the effect of consuming daily 100 g RUTF (ready-to-use therapeutic food) as supplement, on body composition, anemia and zinc status of hospitalized PLWH in Senegal.

**Methods:**

A Controlled clinical trial was conducted in 65 PLWH randomly allocated to receive either standard hospital diet alone (Control group: *n* = 33), or the standard diet supplemented with 100 g RUTF/day (RUTF group: *n* = 32). Supplementation was continued at home during 9 weeks. Individual dietary intakes were measured and compared to the Recommended Dietary Allowances. Body composition was determined using Bio-Impedance Analysis. Hemoglobin was measured by HemoCue and plasma zinc (PZ) concentration by atomic absorption spectrometry. PZ was adjusted to infection (CRP and α1-AGP). All measures were conducted on admission, discharge and after 9 weeks home-based follow up.

**Results:**

34 and 24 % of the patients in RUTF and Control groups were suffering from severe malnutrition (BMI < 16 kg/m^2^), respectively. In both groups, more than 90 % were anemic and zinc deficiency affected over 50 % of the patients. Food consumed by the Control group represented 75, 14 and 55 % of their daily recommended intake (DRI) of energy, iron and zinc, respectively. When 100 g of RUTF was consumed with the standard diet, the DRI of energy and zinc were 100 % covered (2147 kcal, 10.4 mg, respectively), but not iron (2.9 mg). After 9 weeks of supplementation, body weight, and fat-free mass increased significantly by +11 % (*p = 0.033*), and +11.8 % (*p = 0.033*) in the RUTF group, but not in the Control group, while percentage body fat was comparable between groups (*p = 0.888*). In the RUTF group, fat free mass gain is higher in the patients on ART (+11.7 %, *n* = 14; *p = 0.0001)* than in those without ART (+6.2 %, *n* = 6; *p = 0.032*). Anemia decreased significantly with the supplementation, but zinc status, measured using plasma zinc concentration, remained unchanged.

**Conclusion:**

Improving PLWH’ diet with 100 g RUTF for a long period has a positive impact on muscle mass and anemia but not on the zinc status of the patients.

**Trial number:**

NCT02433743, registered 29 April 2015.

## Background

Malnutrition is one of the major complications among PLWH (people living with HIV/AIDS) infection and a significant factor in advancing the disease. Wasting syndrome, closely associated with mortality and morbidity, is defined as a weight loss of at least 10 % in the presence of diarrhea or chronic weakness and documented fever for at least 30 days [1]. HIV-associated wasting was recognized as a negative prognostic indicator and as reported by Tabi & Vogel in Ghana, more patients with HIV/AIDS die because of their poor nutritional status rather than the disease itself [2]. Furthermore, malnourished PLWH have a higher risk of death and suboptimal response to treatment especially when antiretroviral therapy (ART) is initiated [3]. Indeed, studies of body composition have demonstrated that during the wasting syndrome, depleted level of body cell mass, which contains the metabolically active tissue rather than weight loss [4], have been associated with increased risk of death [5, 6]. Wasting in most cases results from a combination of factors, including drug use, medications, concurrent diseases, and HIV itself. However, reduced energy intake is suggested as the primary determinant of HIV-related wasting syndrome [7, 8]. Therefore, there has been considerable interest in the measurement of body composition in patients with HIV/AIDS infection. Such measurements can be useful in conjunction with well-maintained weight records to characterize an individual’s response to various medical or nutritional interventions. Many approaches have been used to reverse weight loss in HIV-infected people, including appetite stimulants, anabolic agents, cytokine inhibitors and hormones [9–11]. In resource-limited settings where malnutrition is the second complication of HIV infection, nutritional intervention programs should be a priority for the successful care of PLWH [12, 13]. In Senegal, we have demonstrated that daily supplementation with 43 g RUTF, a ready to use peanut-based therapeutic food, when mixed with millet porridge provided an additional energy intake of 245 kcal/d, and had a positive impact on body composition, particularly by increasing fat free mass and active cell mass among adults HIV-outpatient with chronic malnutrition [14]. Ndekha et al. in Malawi showed a significant improvement on body mass index (BMI) and fat free mass in adult PLWH supplemented with 245 g RUTF/d providing 1362 kcal, 35.5 g protein, 78 μg of selenium, and 8 mg of zinc [15]. Recently, in South Africa, Evans et al. [16] in a randomized study showed a positive effect of supplementation with a high-protein high-energy meal (100 g equivalent to 388 kcal/d). Others authors have found similar results on body composition using different supplements [14, 17, 18]. In most of these studies, no significant difference was reported on CD4, viral load and quality life. However, the most important observation was that none of these diets did not provide enough zinc to cover the normal zinc requirement of the patients [12, 14, 15, 17], although data from the literature showed that zinc deficiency remains a major problem in PLWH. During HIV infection, zinc deficiency is associated with loss of appetite, disease progression and increased morbidity and mortality [19, 20]. Mocchegiani et al. showed that supplementation with zinc at doses approximately 3 times the RDA significantly increased weight, plasma zinc concentration, CD4 count and reduced the incidence of opportunistic infections in PLWH [21]. World Health Organization (WHO) recommended that at least 1 RDA of zinc should be daily provided to PLWH [22]. In Senegal, the supplementation of PLWH with 43 g RUTF/d covered 86 % of the daily recommended intake of zinc [14]. It is likely that the dose provided daily in this previous study was inadequate (<1 RDA) to cover the zinc requirement of the patients. It is also possible that a low efficiency of zinc absorption from the supplement had occurred due to the presence of phytates in the RUTF and the millet porridge. Millet and RUTF contain a noticeable amount of phytates, and it is now well known that zinc bioavailability is affected by the amounts of phytates consumed with the meal [23, 24]. In view of these above considerations, we decided to increase the daily consumption of RUTF (100 g /d) in order to meet at least one RDA of zinc requirement while maintaining the additional amount of energy consumed as recommended by WHO for PLWH. We also replaced the millet porridge with rice porridge that contains less *myo*-inositol phosphate. Hence, the present study was designed to measure the effect of 100 g/d RUTF consumed with rice porridge on body composition, anemia and zinc status of hospitalized PLWH in Senegal over a 3 month period. We hypothesized that such supplement would significantly improve the nutritional status of PLWH.

## Methods

### Study setting and subjects

The study was conducted from October 2011 to Jun 2012 at the Service des maladies infectieuses (SMI), located in the Centre Hospitalier National Universitaire de Fann (CHNU), Dakar, Senegal (West Africa). Upon admission and clinical examination, the eligibility of the patients enrolled in the study was established as follows: HIV/AIDS men and women at any WHO stages of the disease, under antiretroviral (ART) treatment or not, without psychiatric illness, not diabetic, without long term physical disability, and inability to eat. The study was approved by the Ethical Committee of the Ministry of Health of Senegal and registered with the National Institutes of Health as a clinical trial number NCT02433743. Prior to participation, the patients were informed about the study objectives and procedures, and their written consent was obtained.

### Study design

The study was designed as a randomized, controlled trial with one treatment group and a Control group. Eligible sixty-five (65) HIV- infected adults hospitalized in the SMI were randomly allocated to receive either standard hospital diet (Control group: *n* = 33), or standard hospital diet supplemented with RUTF (RUTF group: *n* = 32). Sample-size calculations was based on the expected difference of 2.3 ± 2.1 kg in change in fat free mass between group from baseline to 9 week, with a 90 % power and 5 % significance level. The enrollment of the patients was carried out by the clinician. The randomization and assignment to group was performed by the senior researcher using a computer-generated random number list (EPI INFO 6.0; Centers for Disease Control and Prevention, Atlanta).

### Diet and nutritional supplement

During hospitalization, the Control group received a standard hospital diet that consisted of three local meals/d (breakfast, lunch and dinner). The meals were provided either by the hospital or by the patient’s relatives. The RUTF group received daily, in addition to the standard hospital diet, 200 g of supplement (100 g RUTF mixed with 100 g of rice porridge). The rice porridge (9.1 g rice flour per 100 ml water) was prepared extemporaneously, mixed with the RUTF, and served immediately. RUTF is composed of peanut butter and skimmed milk powder fortified with a vitamin-mineral complex commercialized by Nutriset. Our laboratory tested RUTF for the first time during the rehabilitation of children suffering from severe and acute malnutrition [13]. All meals were served at 7:00 AM, 1:00 PM and 6:00 PM. The supplement was served twice (2 × 100 g) daily: at 11:00 AM and 5:00 PM. In addition, all the patients (Control and RUTF) received a vegetable-based soup from the micro-garden of the hospital at 12:00 AM.

The nutrient composition of the supplement and percentage coverage of nutrient requirements relative to RDA are shown in Table 1. At discharge, each patient of the RUTF group received 100 g/d RUTF during 9 weeks for home-based supplement preparation. Both groups received dietary counseling to improve their diet at home. Clinical follow-up was performed in both RUTF and Control groups over a 3 months period (during hospitalization, and 9 weeks after). Anthropometry, body composition (fat free mass, fat mass, % body fat), anemia, zinc and infectious status were measured on admission, at discharge, and after 9 weeks follow-up.

**Table 1:** Nutrient composition of the supplement and percentage coverage of daily recommended intake

	Total	Per	%
	Intakes^a^	100 kcal	Coverage^b^
Macronutrients			
Energy kcal	567.7	-	25.3
Protein (g)	14.2	2.5	27.9
Carbohydrate (g)	49.3	8.7	37.9
Lipids (g)	35.8	6.3	130.1
Vitamins			
Vitamin A (μg)	910	160.3	132
Vitamin D (μg)	16	2.8	320
Vitamin E (mg)	20.0	3.5	133.4
Vitamin C (mg)	53	9.3	177.7
Vitamin B1 (mg)	0.6	0.1	53.0
Vitamin B2 (mg)	1.8	0.3	150
Vitamin B6 (mg)	0.6	0.1	47.7
Vitamin B12 (μg)	1.8	0.3	75
Vitamin K (μg)	21	3.7	35
Biotin (μg)	65	11.4	216.7
Folic Acid (μg)	211.8	37.3	53.0
Pantothenic acid (mg)	3.1	0.5	62
Niacin (mg)	5.3	0.9	35.3
Minerals			
Calcium (mg)	320.9	56.5	32.1
Phosphorus (mg)	403.3	71.0	57.6
Potassium (mg)	1119.9	197.3	23.8
Magnesium (mg)	95.2	16.8	39.7
Zinc (mg)	7^c^	1.2	194
Copper (mg)	1.8	0.3	89
Iron (mg)	0.6^d^	0.1	6.2
Iodine (μg)	110	19.4	73.3
Sodium (mg)	189.4	33.4	12.6
Selenium (μg)	30	5.3	100

^a^Supplement =100 g RUTF + 100 g rice porridge

^b^% nutrient coverage calculated using FAO/OMS (2002, 2004)

^c^Zinc intake calculated based on the bioavailability of zinc in mixed diets (Hotz and Brown, 2004)

^d^% iron intake based on the bioavailability of iron (5 %) in the supplement (FAO/OMS, 1989)

### Dietary assessment

Dietary intakes were measured during 7 consecutive days in 10 randomly selected subjects from each group during the hospitalization period. Individual ration of each meal served by the hospital or provided by the relatives was weighed with a food scale (i-Balance 2600 Myweigh, Scale Company, Phoenix, USA). At the end of the meal, the remains (R) were weighed. Thus, the difference between the amount served and the remaining amount was used to calculate the exact amount of food consumed by the patient. Nutrient intakes were calculated using the African food composition tables [25] and Nutrisurvey/ENA software, and were compared to RDA [26, 27].

### Anthropometry

Anthropometric measurements were performed using standard procedures. Measure of height was made using height board (SECA 216, GmbH et Co, Hamburg, Germany), to the nearest millimeter. Body weight was measured with an electronic scale (SECA 877, GmbH & Co, Hamburg, Germany), with a maximal range of 200 kg and a precision of 100 g. The measures were made in duplicate without clothing.

### Body composition

Body composition was measured using Bioelectrical Impedance Analysis (BIA) with a multifrequency analyser (Xitron 4000B, Xitron Technologies, California, USA). The accuracy of the instrument was tested before the measurements by using a 422Ω standard resistor purchased with the analyzer. The resistance and reactance values of measurements are given to the nearest 0.1 Ω from a digital display. Total body water (TBW) was predicted from the equation developed by Diouf et al. in HIV-infected people in Senegal: TBW (kg) = 0.3756(height^2^/R_1000_) + 0.1717(weight) – 0.5756 [28]. Fat free mass, fat mass and % body fat were calculated.

### Blood sampling and analysis

Blood sampling was performed in the morning between 8:00–10:00 AM on admission, at discharge and after 9 week home follow-up. All blood collection and processing procedures were conformed to the recommendations of the International Zinc Nutrition Collaborative Group: IZiNCG [29]. Blood was collected into trace element–free polyethylene zinc-free tubes containing lithium heparin anticoagulant, and centrifuged shortly after. The time of the sample collection and of the most recent food intake were noted and used to adjust for this interval in the data analysis. The reason for this statistical adjustment for the time interval between the last meal and the blood collection was to minimize the variability due to known meal-related effects on plasma zinc concentrations. Hemoglobin concentration was measured by using a HemoCue photometer (HemoCue Hb 201+; HemoCue AB, Angelholm, Sweden). Immunocytometry (Becton Dickinson FACS count Immunocytometry systems, California) determined the CD4 cell count in total blood using FACS count kits reagent (Becton Dickinson, BD Bioscience). Blood samples were centrifuged at 3200 rpm (EBA 20 centrifuge model 2002; Andreas Hettich GmbH & Co KG, Tuttlingen, Germany) during 12 min and plasma samples were collected in trace element-free cryovials and stored at−80 °C in the laboratory until assays. All the analysis was performed in the Laboratory of Nutrition, University Cheikh Anta Diop of Dakar. Plasma zinc (PZ) concentrations were measured in duplicate by atomic absorption spectrophotometry (AA800 Model, Perkin-Elmer Corporation, Norwalk, USA). Quality control of the measurements was performed using the reference material randox assay human serum level 2 (Randox, Antrim, UK). Internal pooled plasma control was run with each batch of sample. Plasma α-1 acid glycoprotein (AGP) and C-reactive protein (CRP) concentrations were quantified by immunoturbidimetry (automate A15, BioSystems S.A, Barcelona, Spain) using BioSystem enzymatic kits (BioSystem).

### Statistical analysis

Statistical analysis was performed using Epi-Info version 3.5.1 (CDC, Atlanta, USA), Excel 2003 (Microsoft Corporation, Redmond, USA) and STATA/SE 11.0 (Stata Corporation, Texas, and USA). Results are expressed as mean ± standard deviation and percentage. Descriptive statistics were used to examine all variables. Baseline characteristics were compared by using student *t*-test for continuous variables and Pearson’s chi-square test for percentage. Linear regression models (for continuous variables) and analysis of variance (for categorical variables) were performed to assess factors associated with plasma zinc concentration. The variables assessed in relation to baseline plasma zinc concentration were as follows: elapsed time from last meal to blood draw, elapsed time from blood draw to centrifugation, elapsed time from centrifugation to separation of plasma, presence of visible hemolysis in the plasma sample, and presence of elevated CRP (≥5 mg/L) and elevated AGP (≥1.0 g/L). Analysis of covariance (univariate General Linear Model procedure) was used to compare changes in weight, BMI, fat free mass and hemoglobin among study groups, and in plasma zinc concentration, after adjustment for baseline plasma zinc concentration, CRP and AGP concentrations, and elapsed time between the previous food intake and the time of the blood sampling for the baseline and final blood draw [29, 30]. All interactions with the main effect were tested for significance, and non-significant variables were removed by using a stepwise procedure. Zinc deficiency was defined according to IZINCG cut-off. *P* values 0.05 were considered as significant.

## Results

**Fig. 1:**
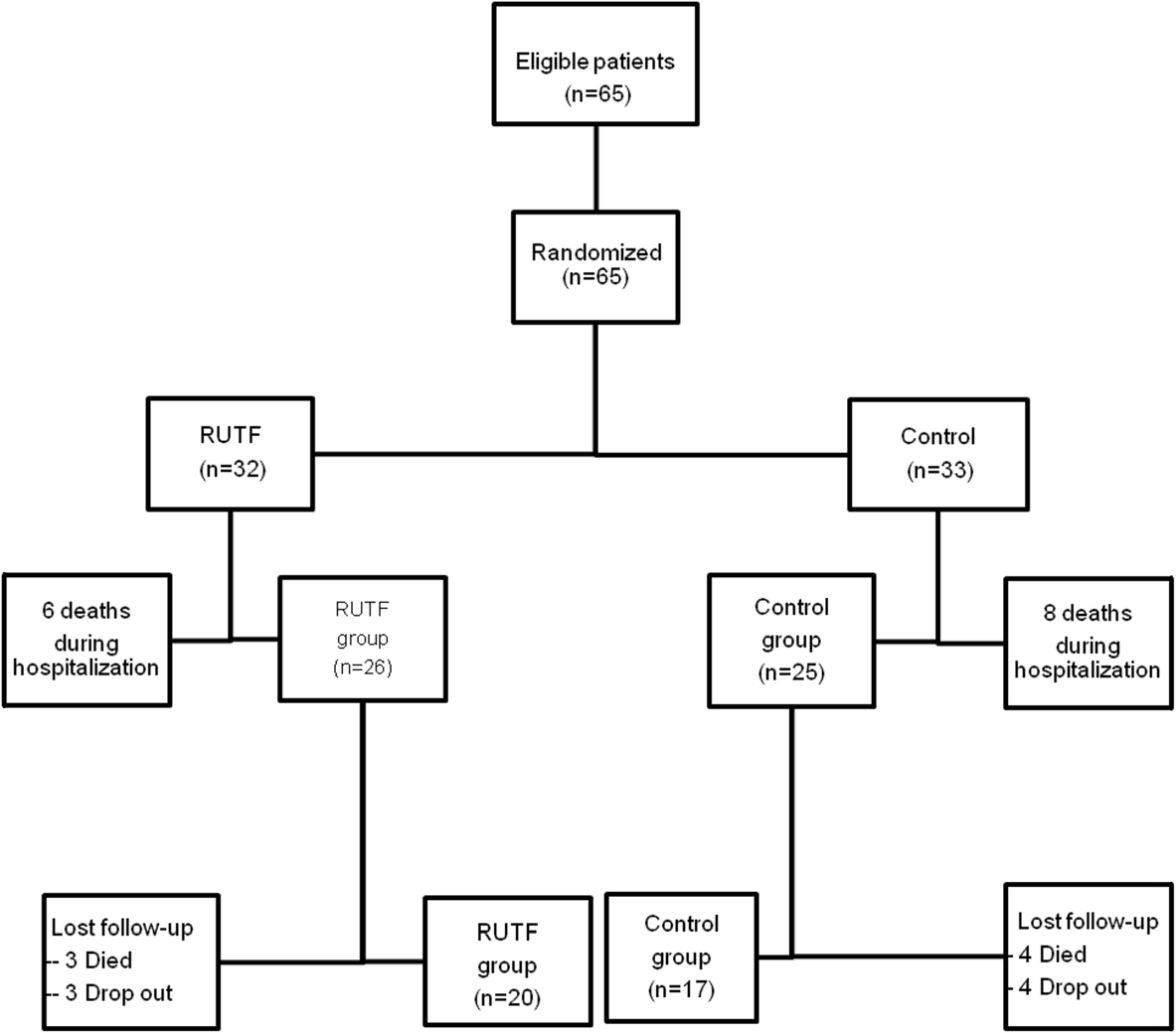
Flow diagram

Sixty-five (65) PLWH patients were randomized into 2 groups: RUTF group (*n* = 32; 24 women and 8 men) and Control group (*n* = 33; 14 women and 19 men). During hospitalization, 6 patients died in the RUTF group and 8 in the Control group. Fifty-one (51) completed the hospital follow up (RUTF group: *n* = 26; and Control group: *n* = 25). After 9 weeks of home monitoring, 3 patients withdrew the study and 3 died in the RUTF group. In the Control group, 4 patients died and 4 were lost during the follow-up. Final analysis concerned 37 patients, 20 in RUTF group and 17 in Control group as shown in the profile of study subjects (Fig. 1). There was any difference in the clinical and anthropometric characteristics between patients who completed the study and those who did not.

### Clinical and nutritional characteristics

At enrollment, majority of patients was serology HIV-1 and had stages three and four according to WHO classification of HIV disease. In each group, over 70 % of the patients were on ART at enrollment, 19/26 and 19/25 patients in the RUTF and Control group, respectively. The median CD4 count was comparable in the RUTF and the Control group (109 ± 137 vs. 128 ± 165 cell/μL; *p = 0.082*). Regardless of the group, tuberculosis was the leading opportunistic infections encountered. Dehydration, chronic diarrhea and oral candidiasis were also present at the initial examination of patients in both groups (Table 2). There were no significant differences for age and weight between the two groups on admission. However, the height was significantly higher in the Control group than in the RUTF group (*p = 0.006*). After adjustment for height, BMI, fat free mass (FFM), fat mass (FM) and percent body fat (%BF) were comparable between the RUTF and the Control groups (Table 3). On admission, 19 (30 %) patients had severe chronic malnutrition (BMI <16.0 kg/m^2^), 11 patients in the RUTF group and 8 in the Control group.

**Table 2:** Clinical and nutritional status of patients at baseline

	Groups		
	Control (*n* = 25)	RUTF (*n* = 26)	*p*
Age (y)	42 ± 12	40 ± 12	0.800
Female, % (n)	52 (22)	86 (13)	0.090
HIV-1, % (n)	84 (21)	92 (24)	0.874
ART, % (n)	76 (19)	73 (19)	1.000
CD4 count),	128 ± 148	109 ± 165	0.082
<200 cells/μL, % (n)	84 (19)	81.7 (21)	0.725.
Weight (kg)	51.4 ± 9.8	47.1 ± 9.4	0.121
Height (m)	1.72 ± 0.08	1.66 ± 0.50	0.006
BMI (kg/m^2^)	17.5 ± 0.6	16.7 ± 0.6	0.711
*< 16 kg/m* ^*2*^ *, % (n)*	24 (8)	34 (11)	0.886
TBW (kg)	8.1 ± 2.7	7.7 ± 2.8	0.121
FFM (kg)	11.1 ± 0.4	10.6 ± 0.4	0.121
BF (kg)	39.4 ± 1.2	37.8 ± 1.2	0.651
% BF	78.0	78.2	0.789
Zinc (μg/dL)	68.1 ± 29.8	68.7 ± 32.4	0.992
Zinc deficiency, % (n)	64 (16)	54 (14)	0.765
Hb (μg/dL)	8.5 ± 2.0	8.4 ± 2.2	0.084
Anemia, % (n)	96 (24)	92 (24)	1.000
Chronic infection, % (n)	32 (8)	31 (8)	1.000

Zinc deficiency: women, PZ <70 g/dL (morning fasting) and PZ < 66 mg/dL (morning nonfasting); men, PZ <74 g/dL (morning fasting) and PZ <70 mg/dL (morning nonfasting)

Anemia: women, Hb <12 μg/dL and men Hb <14 μg/dL

Chronic infection: CRP < 5 mg/L and AGP ≥ 1 g/L

**Table 3:** Energy, zinc, vitamin A and iron intakes and % coverage of daily recommended intake in both groups

	Sex	RDA^a^	Groups			
			Control		RUTF	
			Intakes	% coverage	Intakes^b^	% coverage
Energy(kcal)	Women	2100^c^	1526	73	2108	100
	Men	2520^c^	1946	77	2186	87
Vitamin A (μg)	Women	500	534.7	107	1858	372
	Men	600	1561	260	1828	307
Zinc (mg)^d^	Women	4.9	3.0	61	10.4	212
	Men	7	3.4	49	10.6	151
Iron (mg)^e^	Women	19.6	1.8	9	2.0	10
	Men	9.1	2.3	25	2.9	32

^a^RDA (Recommended Dietary allowances), FAO/WHO [26, 27]

^b^Intakes measured in addition to the supplement (RUTF + rice porridge)

^c^ RDA of Energy is increased by 20 % to take into account the increase of daily recommended intake for PLWH (WHO, [22])

^d^Zinc intake calculated based on the bioavailability of zinc in mixed diets (Hotz and Brown, [50])

^e^Iron intakes calculated by taking into account the bioavailability of iron (15 %)

Initially, mean hemoglobin: 8.5 ± 2.0 vs. 8.4 ± 2.2 (*p* = *0.084*), and plasma zinc concentration: 68.1 ± 29.8 vs. 68.7 ± 32.4 (*p* = *0.992*), were low in the Control and the RUTF group, respectively, but were comparable between groups. Anemia was observed in almost all patients and over 50 % of them were zinc deficient according to IZINC cutoff [29]. More than 30 % of the patients in the Control as well as in the RUTF were suffering from chronic infection defined by CRP < 5 mg/L and AGP ≥ 1 g/L (Table 3).

### Dietary intakes

Initially, the total daily energy intake from the standard hospital diet is low and comparable between groups: 1778 ± 708 kcal vs. 1558 ± 692 kcal in the Control and the RUTF group (*p = 0.503*). Except for vitamin A requirement, the hospital diet associated with the vegetable-based soup was unable to cover the patient’s requirements for iron and zinc (Table 3). By improving the diet with 200 g of supplement (100 g RUTF mixed with 100 g rice porridge), mean daily energy and zinc intakes increased from 1558 to 2147 kcal, and from 3.4 to 10.6 mg zinc in the RUTF group, reaching 100 % of requirements for both nutrients. The supplement also improved the daily intakes of vitamins C, D, E and vitamins B complex. However, the iron intake covered only 1/3 of patients’ needs (Table 3).

### Effect of the supplement on body composition

No difference was found in the hospital length between the Control and the RUTF group: 27 ± 18 and 20 ± 10 days (*p* = *0.114*), respectively. At discharge, clinical and nutritional parameters were comparable in both groups (Table 4). But, after 9 weeks home-based supplementation body weight, BMI, fat free mass, fat mass, hemoglobin were significantly higher (*p < 0.05*) in the RUTF group than in the Control group (Table 4). ANOVAs analysis showed that consumption of 100 g RUTF for 3 months significantly increased body weight (+11 %; *p = 0.033*), fat free mass (+11.8; *p* = *0.033*), fat mass (+10.7 %; *p* = *0.032*) and decreased body fat percentage (*p < 0.05*) compared to the non-supplemented group. In the supplemented group, fat free mass increased significantly more in the patients on ART (+11.7 %, *n* = 14; *p = 0.0001*) than in those who did not received ART (+6.2 %, *n* = 6; *p = 0.032*).

**Table 4:** Changes in body composition and biological parameters from baseline to 9 weeks in both groups

	Baseline			Discharge from hospital			9 weeks		
	Control (*n* = 25)	RUTF (*n* = 26)	*p*	Control (*n* = 25)	RUTF (*n* = 26)	*p*	Control (*n* = 17)	RUTF (*n* = 20)	*p*
Weight (kg)	51.4 ± 9.8	47.1 ± 9.4^a^	0.121	50.5 ± 9.6	49.3 ± 9.1	0.651	49.7 ± 8.6	54.2 ± 8.9^b^	0.009
BMI (kg/m^2^)	17.5 ± 0.6	16.7 ± 0.6^a^	0.711	17.3 ± 0.6	17.6 ± 0.6	0.445	16.6 ± 0.7	19.3 ± 0.6^b^	0.009
TBW (kg)	8.1 ± 2.7	7.7 ± 2.8^a^	0.121	7.9 ± 0.3	8.1 ± 0.3	0.650	7.7 ± 0.3	8.9 ± 0.3^b^	0.011
FFM (kg)	11.1 ± 0.4	10.6 ± 0.4^a^	0.121	10.8 ± 0.4	11.1 ± 0.4	0.650	10.5 ± 0.5	12.3 ± 0.4^b^	0.011
BF (kg)	39.4 ± 1.2	37.8 ± 1.2^a^	0.651	38.5 ± 1.4	39.4 ± 1.4	0.651	37.5 ± 1.5	43.4 ± 1.4^b^	0.009
% BF	78.0	78.2^a^	0.789	78.1	78.1	1.000	78.1	77.9^b^	0.888
Zinc (μg/dL)	68.1 ± 29.8	68.7 ± 32.4	0.992	72.6 ± 41.3	59.0 ± 24.2	0.220	71.6 ± 19.4	74.3 ± 45.3	0.811
Zinc deficiency, % (n)	64 (16)	54 (14)	0.765	44 (11)	65 (17)	0.187	41 (7)	65 (13)	0.147
Hb (μg/dL)	8.5 ± 2.0^d^	8.4 ± 2.2^a^	0.844	9.7 ± 1.7	9.9 ± 1.9^b^	0.649	10.0 ± 1.7^e^	11.8 ± 1.5^c^	0.002
Anemia, % (n)	96 (24)	92 (24)^a^	1.000	88 (21)	77 (20)	0.502	94 (16)	50 (10)^b^	0.003

Groups mean were compared by student *t*-test (Control vs. RUTF group)

Zinc deficiency: women, PZ <70 g/dL (morning fasting) and PZ < 66 mg/dL (morning nonfasting); men, PZ <74 g/dL (morning fasting) and PZ <70 mg/dL (morning nonfasting); Anemia: Hb <12 μg/dL (women) and <14 μg/dL (men)

^a, b, a/c^ and ^b/c, d/e^ (*p < 0.05*): ANOVA for repeated measures with Bonferroni correction within group

### Effect of supplement on anemia and zinc status

After 9 weeks, patients in RUTF and Control groups showed improvement in their hemoglobin level, but the change in hemoglobin was significantly higher in the RUTF than in the Control group: +3.33 ± 0.63 μg/dL and +0.82 ± 0.49 μg/dL, respectively (*p = 0.004*). Therefore, the number of anemic patients decreased significantly in the RUTF group (94 to 50 %; *p = 0.003*) while it remained unchanged in the Control group (96 to 94 %). Contrary to hemoglobin, the mean change in plasma zinc concentration was comparable between the RUTF group and the Control group (*p =0.811*). Mean plasma zinc concentrations in both groups did not differ from their respective baseline values (Table 4).

## Discussion

The present randomized control study was designed to evaluate the effect of consuming daily 100 g RUTF mixed with rice porridge as supplement on body composition, anemia and zinc status of hospitalized PLWH in Senegal during hospitalization and after 9 weeks home-based supplementation. A high prevalence of severe chronic malnutrition was detected among the Senegalese PLWH, and according to several authors [14, 15], the nutritional quality of the patients’ diets seems to be the immediate cause of this high prevalence of malnutrition, even thought others causes like decrease food consumption due to several factors, especially anorexia accompanying opportunistic infections, may be suspected [8]. In this study, the standard hospital diet as well as diets provided by the relatives, even fortified with a vegetable soup was unable to cover the patients’ needs. Improving the diet with a high-energy dense fortified supplement, consisting of 100 g RUTF mixed with rice porridge improved significantly the diet in term of energy, vitamins (B complex vitamins except for biotin, C, D, E, and minerals intakes (zinc, selenium, cooper, calcium, potassium, phosphorus, magnesium). Low serum levels of calcium, potassium, and magnesium have been reported in HIV-infected population [31–33]. In contrast, high plasma concentration of copper was founded early in the course of HIV infection [34], due to the increase of this mineral during the acute-phase response in a variety of infections and inflammatory conditions. However, the effect of supplementation with these minerals on HIV population was poorly investigated and requires further research.

The daily recommended intake of vitamin A in our patients was covered by consumption of a vegetable-based soup prepared by the hospital and rich in beta-carotene. But we did not investigated the others nutrients in the soup. The home diet of the patients was not measured but it is expected the vitamin A stores gained during hospitalization remains for several weeks.

This was not the case for iron. During hospitalization, iron intake from the patients’ diet was very low as well as the iron content of 100 g RUTF. Although the supplementation increased a bit the iron intake of the RUTF group, it did not reach the daily recommended intake, and all the positive effects on the hemoglobin level in this group cannot be assigned to iron intake. In a previous study from our laboratory (unpublished data), we showed that Senegalese outpatients PLWH with anemia were not iron deficient according to their plasma ferritin concentration or serum transferrin receptor. In the present study, the prevalence of anemia was and remained high after 9 weeks follow-up in both groups, and these results are similar to those reported by others [14, 16, 35]. The failure of erythropoiesis is generally considered as the primary physiopathological mechanism of anemia in HIV-associated opportunistic infections, micronutrient deficiencies, direct effect of HIV on erythropoiesis and tuberculosis mediated-anemia [36–38]. The positive effect of the RUTF supplementation on hemoglobin and the significant reduction of the anemic patients in the RUTF group at 9 weeks were not correlated with their iron intake. It is reported that iron supplementation have a negative effect on HIV-infection [39]. In our study, the iron intake from the supplement was very low (2.9 mg/day) and was not expected to have similar negative affects when high dose of iron (30 to 60 mg/day) were administrated. This result suggests an anabolic effect of other micronutrients from the RUTF, perhaps increased intakes of B12 and folic acid. The positive effect of vitamins B complex on wasting has been demonstrated by several authors [40, 41]. Irlam et al., in the recent Cochrane review showed that multivitamin supplementation trial increased weight gain in PLWH co-infected with TB and HIV [40]. These results of vitamins on wasting could be related to their effects at various levels of immunity [42, 43]. Furthermore, recent studies support beneficial effects of micronutrient supplementation (vitamin B complex, vitamin C and E) at RDA level in HIV-infected people [44, 45].

At the end of the hospitalization period, any beneficial effect of the supplementation was observed on body composition but after 9 weeks home supplementation, the change in body composition was highly significant from baseline (+11 % body mass). The increase in fat free mass in the RUTF group could be explained by the increase coverage of certain nutrients, particularly vitamins and minerals. It could also result from the reduction of opportunistic infections during hospitalization. Infectious episodes are accompanied by increase protein catabolism which partially explains the high rate of severe malnutrition observed in our patients in both groups, and their low initial fat free mass. Also, multivitamins could directly avert muscle loss by decreasing the oxidative stress that has been found to result in muscle wasting [42, 43]. However, in the present study, reduction of .opportunistic infections was observed in the RUTF as well as in the Control, since it was the discharge criteria from the hospital. Therefore, it is more likely that the significant improvement in body composition in the RUTF group compared to the Control group can be assigned to the supplement. Increase of fat free mass has been reported by others authors even with lower dose of RUTF [14, 15]. Diouf et al. in a study on adults outpatients PLWH supplemented with 43 g of RUTF per day showed an increase in fat free mass of about 7.7 % after 6 months of supplementation [14]. With higher dose of RUTF (245 g of RUTF/d) over a period of 3.5 months, Ndekha et al. also demonstrate an increase in lean mass, but, in contrast with our study, food consumption was not measured [15]. In the present study, the percentage increase in fat free mass after 3 months of supplementation was 11.8 % indicating that an unbalance diets supplemented with 100 g RUTF can lead to a significant positive effect on muscle mass without increasing the % body fat. These results suggested that the energy intake of 100 g RUTF (an energy dense fortified food) is sufficient to optimize the use of other nutrients that causes a significant gain in body mass, probably because the amount of energy supplied by 100 g RUTF (>500 kcal). It is well known that energy density is a determinant of food consumption [46] and can increase the chances of nutritional recovery of severely malnourished HIV-infected patients in sub Saharan Africa with BMI less than 17 kg/m^2^ [47]. Yet, the vitamin/mineral content of 100 g RUTF as food complement needs more investigations according to local diet habits. We showed in the present study that such quantity is well accepted by PLWH and has positive effect on their nutritional status.

The prevalence of zinc deficiency was high (50 %) in both groups although the supplement had covered the zinc requirements in the RUTF group. High prevalence of zinc deficiency in PLWH has been reported by other authors [20, 48]. In the present study, zinc intake was increased with the supplementation to more than one RDA of zinc as recommended by WHO [22]. RUTF is a zinc-fortified food but the fortification was not reflected on the plasma zinc concentration of the patients. This result suggested that either one RDA is not enough to cover the zinc requirement of PLWH, or the concentration of plasma zinc does not reflect zinc status during fortification contrary to zinc supplementation. In our laboratory, we have demonstrated that plasma zinc concentration increased in children and adults who received daily zinc supplementation for a short time but not in those who received a zinc-fortified food containing a similar amount of zinc [24, 49]. Also, Mocchegiani et al. showed that zinc supplementation with 3 times the RDA increases significantly the plasma zinc of PLWH [21]. The question remains open and needs additional studies to assess the usefulness of plasma zinc as biomarker of zinc fortification. Moreover, the disturbance in the homeostasis of zinc, particularly, the redistribution of zinc to the liver and the presence of inflammatory cytokines or proteins may be implicated in the etiology of zinc deficiency among PLWH as well as the use of zinc for the expression of genes and the replication of HIV [50].

## Conclusion

Severe chronic malnutrition is highly prevalent among Senegalese hospitalized PLWH with a significant loss of fat free mass. The patient’s usual diet did not cover most of the nutrient requirements, including energy. Improving this diet with a supplement consisting of 100 g RUTF (a high energy dense fortified food) mixed in rice allowed to cover the RDA of many micronutrients including zinc for PLWH. The supplement had a positive effect on body composition by increasing the amount of lean and fat mass and maintaining % body fat. Within the supplemented group, food + treatment were better at restoring fat free mass and resolving anemia than treatment alone. The anemic status of the patients was also significantly improved despite a low iron intake. It is possible that the supplement has improved the zinc status of the PLWH (fat free mass deposit increased), but we were unable to confirm such improvement as their plasma zinc concentration failed to reflect it. Research is needed to identify more sensitive indicator of zinc status in PLWH in response to zinc fortification. However, from the overall results, this study, we would like to recommend supplementation of adult PLWH with 100 g RUTF in the early stage of the infection.

## Abbreviations

ART: Antiretroviral treatment
BIA: Bio-Impedance Analysis
CHNU: Centre Hospitalier National Universitaire
HIV: Human Immunodeficiency Virus
PLWH: People Living With HIV/AIDS
RDA: Recommended Dietary Allowance
RUTF: Ready-to-Use Therapeutic Food
WHO: World Health Organization
